# Effect of *EPSPS* gene copy number and glyphosate selection on fitness of glyphosate-resistant *Bassia scoparia* in the field

**DOI:** 10.1038/s41598-021-95517-2

**Published:** 2021-08-09

**Authors:** Charlemagne Ajoc Lim, Prashant Jha, Vipan Kumar, Alan T. Dyer

**Affiliations:** 1 Department of Plant Sciences and Plant Pathology, Montana State University, Bozeman, MT USA; 2 Department of Agronomy, Iowa State University, Ames, IA USA; 3 Agricultural Research Center, Kansas State University, Hays, KS USA

**Keywords:** Ecology, Plant sciences

## Abstract

The widespread evolution of glyphosate-resistant (GR) *Bassia scoparia* in the U.S. Great Plains poses a serious threat to the long-term sustainability of GR sugar beet. Glyphosate resistance in *B. scoparia* is due to an increase in the *EPSPS* (5-enolpyruvyl-shikimate-3-phosphate) gene copy number. The variation in *EPSPS* gene copies among individuals from within a single GR *B. scoparia* population indicated a differential response to glyphosate selection. With the continued use of glyphosate in GR sugar beet, the effect of increasing glyphosate rates (applied as single or sequential applications) on the fitness of GR *B. scoparia* individuals with variable *EPSPS* gene copies was tested under field conditions. The variation in *EPSPS* gene copy number and total glyphosate rate (single or sequential applications) did not influence any of the reproductive traits of GR *B. scoparia*, except seed production. Sequential applications of glyphosate with a total rate of 2214 g ae ha^−1^ or higher prevented seed production in *B. scopari*a plants with 2–4 (low levels of resistance) and 5–6 (moderate levels of resistance) *EPSPS* gene copies. Timely sequential applications of glyphosate (full recommended rates) can potentially slow down the evolution of GR *B. scoparia* with low to moderate levels of resistance (2–6 *EPSPS* gene copies), but any survivors (highly-resistant individuals with ≥ 8 *EPSPS* gene copies) need to be mechanically removed before flowering from GR sugar beet fields. This research warrants the need to adopt ecologically based, multi-tactic strategies to reduce exposure of *B. scoparia* to glyphosate in GR sugar beet.

## Introduction

Kochia [*Bassia scoparia* (L.) A. J. Scott] is one of the most troublesome summer annual broadleaf weed species in croplands and noncroplands across North American Great Plains^1^. *B. scoparia* possesses several unique biological attributes, including early and extended period of emergence, low seed persistence, aggressive growth, and tolerance to various biotic and abiotic stresses, prolific seed production, and tumble mechanism of seed dispersal^1–3^. *B. scoparia* is a monoecious plant species with a protogynous flowering that manifests a high outcrossing and pollen-mediated gene flow within and among field populations^4,5^. Consequently, *B. scoparia* is a genetically diverse weed species, with a high tendency to evolve herbicide resistance^5,6^.

*Bassia scoparia* is ranked among top six most problematic weeds of sugar beet (*Beta vulgaris* L.)^7,8^. Sugar beet is particularly sensitive to early-season competition from *B. scoparia* that may result in significant beet root yield reductions (up to 95%)^9–11^. In general, *B. scoparia* is more difficult to control in sugar beet than in corn (*Zea mays* L.), soybean [*Glycine max* (L.) Merr.], wheat (*Triticum aestivum* L.) or fallow^12^.Limited herbicide options and widespread occurrence of *B. scoparia* populations with resistance to triflusulfuron (ALS inhibitor) in conventional sugar beet has led to a rapid adoption of GR sugar beet in the U.S.^13,14^. Two to three applications of glyphosate have been a common practice for weed control in GR sugar beet^14^. Ironically, several GR *B. scoparia* populations have been reported in the Great Plains region since its first discovery in wheat-fallow systems in the western Kansas in 2007^15^. Currently, GR *B. scoparia* from sugar beet fields have been reported from Montana, Wyoming, Nebraska, Colorado, Idaho and Oregon^6,16^. These reports on GR *B. scoparia* from sugar beet fields pose a serious threat to the long-term sustainability of GR sugar beet technology.

Glyphosate resistance in *B. scoparia* has evolved through amplification of the target gene that encodes the 5-enolpyruvyl-shikimate-3-phosphate synthase (*EPSPS)* enzyme (a key enzyme of the shikimate pathway)^15,17,18^. It has been proposed that amplified *EPSPS* copies are generally arranged in tandem in GR *B. scoparia* genome and a mobile genetic element with a FHY3/FAR1-like gene is responsible for the origin of the *EPSPS* gene duplication event and the evolution of glyphosate resistance^19,20^. Previous studies have also shown that increased *EPSPS* copies in GR *B. scoparia* correlate positively with higher *EPSPS* transcription, EPSPS protein, and glyphosate resistance levels^16–19^.

Predicting the pleiotropic effects of *EPSPS* gene amplification and overexpression on fitness attributes of any GR plant species can be challenging^21^. Fitness costs associated with *EPSPS* gene amplification in GR *B. scoparia* have previously been studied in the absence of glyphosate and shown mixed results. For instance, a greenhouse study using inbred lines of GR and glyphosate-susceptible (GS) *B. scoparia* exhibited no differences in growth and reproductive parameters under intraspecific competition^22^. However, the GR *B. scoparia* has shown reduced seed longevity, slower germination rate, and less total germination than the GS *B. scoparia* in other studies^23,24^. Additionally, a competitive greenhouse study using segregating F2 populations of GR *B. scoparia* indicated that plants with higher *EPSPS* copy numbers had reduced seed count and weight, reduced competitive ability, and reduced final height in mixed stands^25^. However, the observed growth and reproductive parameters were highly variable and fitness consequences varied with the genetic background^25^.

Considerable variation in *EPSPS* gene copy number (3 to 13 copies) among individuals from within a single GR *B. scoparia* population suggests a differential response among individuals to varying glyphosate rates^22^. Considering the continued use of GR sugar beet technology in conjunction with extensive use of glyphosate for broad-spectrum weed control, it is crucial to understand the impact of glyphosate applications on the population dynamics of GR *B. scoparia* population with variable *EPSPS* gene copy numbers. Hence, the main goal of this research was to determine the survival (% control) and reproductive characteristics of GR *B. scoparia* with low to high levels of resistance (based on *EPSPS* gene copy numbers) in response to varying glyphosate rates under field conditions.

## Results

The year-by-treatment interaction was not significant for any of the observed response variables in the field study; therefore, all data were combined across the two years. A differential response of *B. scoparia* plants with varying *EPSPS* copy numbers was observed across glyphosate rates tested. The *B. scoparia* plants with ≥ 2 *EPSPS* gene copies survived the field-use rate (870 g ae ha^−1^) of glyphosate, but plants with 1 *EPSPS* gene copy number did not survive. Therefore, except for percent visible control, other response variables were not obtained for plants with one *EPSPS* gene copy number (GS) treated with ≥ 870 g ae ha^−1^ of glyphosate.

### Effect of *EPSPS *gene copy number on reproductive traits of *B. scoparia* in the absence of glyphosate

In the absence of glyphosate, the *EPSPS* gene copy number had no effect on time to 50% flowering (*P* = 0.17), time to set seeds (*P* = 0.18), pollen viability (*P* = 0.23), seeds plant^-1^ (*P* = 0.94), and seed viability (*P* = 0.41), but had a significant effect on 1000-seed weight and seedling (progeny) radicle length (*P* < 0.01) (Table 1). The averaged 1000-seed weight of GR *B. scoparia* plants (2–4, 5–6 and ≥ 8 *EPSPS* gene copies) were lower (0.60 g) compared to the averaged 1000-seed weight of GS plants with 1 *EPSPS* gene copy (0.85 g) at Tukey–Kramer’s HSD_*α(0.05)*_ = *0.09*. Similarly, the averaged seedling radicle length from progeny seeds of GR *B. scoparia* plants were lower (0.45 cm) compared to the averaged seedling radicle length from progeny seeds of GS plants (0.74 cm) at Tukey–Kramer’s HSD_*α(0.05)*_ = *0.17*. These results suggest that GS *B. scoparia* plants (those with 1 *EPSPS* gene copy) may be more competitive than the GR plants in the absence of glyphosate. Nevertheless, the high outcrossing nature of *B. scoparia* and the flow of resistance genes facilitated through shedding of equally viable pollens from resistant plants may contribute to sufficient number of GR individuals in field populations, even if the use of glyphosate is discontinued.

**Table 1:** *F* statistics and *P* values for the effect of *EPSPS* gene copy number on reproductive traits of *Bassia scoparia* plants in the absence of glyphosate in the field averaged over 2 years at the Montana State University Southern Agricultural Research Center near Huntley, MT, USA.

Reproductive traits	*F*	*P*
Time to flowering	1.75	0.17
Time to set seeds	1.70	0.18
Pollen viability	1.52	0.23
Seeds plant^-1^	0.13	0.94
1000-seed weight	40.3	< 0.01
Seed viability	0.97	0.41
Radicle length	12.9	< 0.01

### Effect of *EPSPS *gene copy number and glyphosate on control and reproductive traits of GR. *B. scoparia*

#### Visible control

The *EPSPS* gene copy number, total glyphosate rate (applied as single or sequential applications), and their interactions influenced *B. scoparia* percent visible control (P < 0.01, Table 2). A single application of glyphosate (1265 g ae ha^−1^) provided poor (11%) control of GR *B. scoparia* plants with 2–4 *EPSPS* gene copies, and no control of *B. scoparia* plants with 5–6 or ≥ 8 *EPSPS* gene copies. Two sequential applications of glyphosate (a total of 1740 g ae ha^−1^) provided 72% control of GR plants with 2–4 *EPSPS* gene copies, 36% control for plants with 5–6 *EPSPS* gene copies, and < 5% control for GR plants with ≥ 8 *EPSPS* gene copies. Two sequential applications of glyphosate (a total of 2214 g ae ha^−1^) had 98% and 95% control of GR plants with 2–4 and 5–6 *EPSPS* gene copies, respectively; however, control was poor (< 10%) for plants with ≥ 8 *EPSPS* gene copies. Even with four sequential applications of glyphosate (a total of 3954 g ae ha^−1^, maximum labeled use rate of glyphosate in GR sugar beet in a growing season), control of GR *B. scoparia* with ≥ 8 *EPSPS* gene copies averaged 31% only. Glyphosate rate-response analysis indicated that the total glyphosate rate needed to achieve 90% control (*ED*_90_ values) was 1971 and 2123 g ae ha^−1^ for GR *B. scoparia* with 2–4 and 5–6 *EPSPS* gene copies, respectively (Table 3). For GR *B. scoparia* with ≥ 8 *EPSPS* gene copies, the total rate of glyphosate needed to achieve 90% control was estimated to be > 4000 g ae ha^−1^, indicating very high resistance and might not be controlled if present in GR sugar beet.

**Table 2:** *F* statistics and *P* values for the effects of *EPSPS* gene copy number (2–4, 5–6, and ≥ 8 *EPSPS* gene copies), total glyphosate rate (870, 1265, 1740, 2214, 3084, and 3954 g ae ha^−1^ applied as single or sequential applications), and their interactions on percent visible control, reproductive traits, and relative fitness of glyphosate-resistant *Bassia scoparia* plants in the field averaged across 2 years at the Montana State University Southern Agricultural Research Center near Huntley, MT, USA.

Control and reproductive traits	*EPSPS *copy number		Total glyphosate rate		Interaction	
	*F*	*P*	*F*	*P*	*F*	*P*
Percent visible control	5284	< 0.01	3380	< 0.01	455	< 0.01
Seeds plant^−1^	164.5	< 0.01	83.50	< 0.01	38.36	< 0.01
1000-seed weight	2.55	0.084	0.27	0.93	0.14	0.96
Time to set seeds	0.82	0.44	0.58	0.72	0.45	0.77
Pollen viability	3.00	0.075	0.30	0.87	2.04	0.16
Seed viability	0.62	0.54	1.00	0.42	3.04	0.22
Radicle length	0.07	0.93	0.61	0.69	0.63	0.64
Relative fitness (*w*)	683.3	< 0.01	581.2	< 0.01	113.4	< 0.01

**Table 3:** Estimated parameters (b = slope, c = lower limit, d = upper limit, e = inflection point [ED50 or SR50]) from a four-parameter log-logistic model used to describe the percent visible control and seed production (no. plant^−1^) of *Bassia scoparia* with varying *EPSPS* gene copies (1, 2–4, 5–6, and ≥ 8) in the presence of glyphosate applied at 0, 108, 217, 435, 870, 1265, 1740, 2214, 3084, and 3954 g ae ha^−1^ in the field averaged over 2 years conducted at the Montana State University Southern Agricultural Research Center near Huntley, MT, USA.

*EPSPS* gene copy number	Parameter estimate^d^				
	*b*	*c*	*d*	*ED*_50_ (95% CI)^a,b^	*ED*_90_ (95% CI)^a,b^
**Visible control (%)**					
1	−1.8 d	0.1 a	101.5 a	127 (123–131) d	426 (395–456) d
2–4	−10.3 b	−0.0 a	100.0 a	1594 (1581–1606) c	1971 (1944–1997) c
5–6	−13.8 a	−0.14 a	99.6 a	1810 (1799–1822) b	2123 (2084–2161) b
≥ 8	−4.1 c	−0.14 a	49.8 b	3520 (2881–4159) a	5991 (4152–7830) a

*EPSPS* gene copy number	Parameter estimate^d^				
	*b*	*c*	*d*	*SR*_50_ (95% CI)^a,c^	*SR*_99_ (95% CI)^a,c^
**Seeds plant**^**−1**^** (% of nontreated)**					
1	5.9 b	−0.09 a	97.1 a	290 (273–306) d	520 (456–584) c
2–4	8.5 ab	−0.36 a	99.9 a	1263 (1232–1294) c	1902 (1695–2109) b
5–6	11.6 a	−0.81 a	98.0 a	1577 (1539–1616) b	2130 (2014–2214) b
≥ 8	2.0 c	−0.00 a	99.2 a	13,670 (11,892–15,447) a	23,021 (18,720–24,762) a

^a^Values in parenthesis represent the 95% confidence interval of the respective parameter estimate.

^b^ED_50_ or ED_90_ are estimated glyphosate rates (g ae ha^−1^) required to achieve 50% or 90% visible control, respectively.

^c^SR_50_ or SR_99_ are the estimated glyphosate rates (g ae ha^−1^) required to achieve 50% or 99% reduction in seed production, respectively.

^d^ED_50_, ED_90_, SR_50_ or SR_99_ estimates followed by the same letter are not different based on an approximate t-test using the “CompParm” and “EDcomp” functions in the drc package, R software^38^.

#### Seed production

The *EPSPS* gene copy number, total glyphosate rate (applied as single or sequential applications), and their interactions influenced GR *B. scoparia* seed production (*P* < 0.01, Table 2). Glyphosate rate-response analysis indicated that the total glyphosate rate needed for 99% seed reduction (SR_99_ values) was 1902 and 2130 g ae ha^−1^ for GR plants with 2–4 and 5–6 *EPSPS* gene copies, respectively (Table 3). The SR_99_ value for GR *B. scoparia* plants with ≥ 8 *EPSPS* gene copies were estimated to be > 4000 g ae ha^−1^ of glyphosate. This indicates that seed production in highly resistant plants will not be prevented even with four sequential applications of the maximum-labeled use rate of glyphosate (total of 3954 g ae ha^−1^ of glyphosate) in a GR sugar beet field. However, seed production of GR *B. scoparia* plants with 2–4 (low-level resistance) and 5–6 (moderate level resistance) *EPSPS* gene copies that emerge before the 2-leaf stage of sugar beet can be prevented with three sequential applications of glyphosate, with a total rate of 3084 g ae ha^−1^. However, any survivors may increase the risk of GR progenies with higher levels of evolved resistance in the population.

#### Other reproductive traits

For GR *B. scoparia* plants, the variation in *EPSPS* gene copy number (those with 2–4, 5–6, ≥ 8 *EPSPS* gene copies), total glyphosate rate (applied as single or sequential applications), and their interactions did not influence time to 50% flowering, time to seed set, pollen viability, 1000-seed weight, seed viability, and seedling radicle length (Table 2). These results indicate that seedling vigor of progenies from GR *B. scoparia* plants (2 to ≥ 8 *EPSPS* gene copies) will be comparable regardless of the glyphosate rate, and if let to set seed can potentially build up resistance very rapidly due to pollen-mediated gene flow. The range for time to 50% flowering was 39–41 days after transplanting, time to set seeds was 57–60 days after transplanting, pollen viability was 82–86%, 1000-seed weight was 0.59–0.61 g, seed viability was 94–99%, and radicle length was 0.53–0.54 cm across *EPSPS* gene copy numbers and total glyphosate rates.

#### Relative fitness

Variation in *EPSPS* gene copy number (2–4, 5–6, and ≥ 8 *EPSPS* gene copies), total glyphosate rate (applied as single or sequential applications), and their interactions had a significant effect on the relative fitness of GR *B. scoparia* (Table 4). In the presence of glyphosate applied at total rates ≥ 2214 g ae ha^−1^, GR *B. scoparia* plants with 2–4 and 5–6 *EPSPS* gene copies were not fit (both had *w* = 0; failed to produce offspring) (Table 4). Although four sequential applications of glyphosate (total rate of 3954 g ae ha^−1^) provided little control (31%) of *B. scoparia* plants with ≥ 8 *EPSPS* gene copies, there was a significant reduction in their relative fitness (*w* = 0.46) compared to their fitness at a single application of 870 or 1265 g ae ha^−1^ (*w* = 0.74 and* w* = 0.73, respectively) (Table 4). The results imply that in the presence of glyphosate, GR plants will produce less number of offspring than the most successful genotype (susceptible *B. scoparia* plants with 1 EPSPS gene copy and a *w* of 1.0 in the absence of glyphosate). Selection coefficient (*s* = 1 − *w*) is the measure of the relative strength of the selection agent acting against a genotype^26^. The selection coefficient was 1.0 when glyphosate was applied sequentially with a total of at least 2214 g ae ha^−1^ on GR plants with 2–4 or 5–6 *EPSPS* gene copies, implying that the magnitude of fitness reduction of GR plants with low to moderate levels of resistance was 100% at those glyphosate rates in the field.

**Table 4:** Relative fitness (w) of glyphosate-resistant (GR) *Bassia scoparia* plants (2–4, 5–6, ≥ 8 *EPSPS* gene copies) in the absence and presence of glyphosate (total rate ≥ 870 g ae ha^-1^ applied as single or sequential applications) in a field study conducted over two years at the Montana State University Southern Agricultural Research Center near Huntley, MT, USA.

Total glyphosate (g ae ha^−1^)	Relative fitness (*w*)^a,b^		
	GR plants with 2–4 *EPSPS* gene copies	GR plants with 5–6 *EPSPS* gene copies	GR plants with ≥ 8 *EPSPS* gene copies
0	0.79 a	0.80 a	0.78 a
870	0.76 ab	0.76 ab	0.74 ab
1265	0.34 cd	0.70 ab	0.73 ab
1740	0.04 e	0.20 d	0.69 ab
2214	0.00 e	0.00 e	0.67 ab
3084	0.00 e	0.00 e	0.63 b
3954	0.00 e	0.00 e	0.46 c

^a^Relative fitness was calculated as the reproductive rate (seeds plant^−1^) of the resistant genotype relative to the maximum reproductive rate of the susceptible genotype in the population. The relative fitness (*w*) of the susceptible plants was 1.0.

^b^Means followed by the same letter are not different based on Tukey–Kramer’s HSD_*α(0.05)*_ = *0.135*.

## Discussion

This field study evaluated the reproductive fitness of *B. scoparia* with variable *EPSPS* gene copy numbers to increasing glyphosate rates applied as single or sequential applications, simulating glyphosate applications in GR sugar beet. The GS and GR *B. scoparia* sub-populations were derived from within a single field population; hence, expected to have similar genetic backgrounds^21,22,25^. Fitness consequences of glyphosate resistance alleles in weed species may vary across genetic backgrounds^25^. This is more complicated for *B. scoparia* with a high genetic diversity due to pollen and seed mediated gene flow accompanied by tumble mechanism of seed dispersal within and among fields^4–6^. Furthermore, the GR and GS subpopulatons were subjected to three generations of recurrent group selection which allowed us to use relatively homozygous plants for this fitness study. Seedlings from the selected GR *B. scoparia* subpopulation showed a variable level of glyphosate resistance evident from two to ≥ 8 *EPSPS* gene copies, conferring low to high levels of resistance.

Results indicated that a discontinuity of glyphosate use would favor GS (one *EPSPS* gene copy number) over GR *B. scoparia* because of a greater progeny seedling vigor (higher 1000-seed weight and radicle length) of the former in the absence of glyphosate. The GS individuals would be at a competitive advantage over GR individuals through an earlier establishment and a more vigorous growth in the absence of glyphosate in the field. These findings are consistent with Martin et al.^25^, who reported that progeny seed weights of GR *B. scoparia* plants (10 *EPSPS* gene copies) from Canada were less compared to GS plants (one *EPSPS* gene copy) in a greenhouse study. However in this field study, there were no differences in other reproductive traits (time to flowering, time to set seeds, pollen viability, seeds plant^−1^, or seed viability) between GR and GS *B. scoparia*. Fitness consequences may vary depending on life history stages^24^. Also in previous studies (field and greenhouse), no fitness differences were observed between GR and GS *B. scoparia* in their vegetative growth or seed production but GR individuals had a lower total germination and a slower germination rate than GS individuals^22,24^. Delayed germination and less seedling vigor and subsequent early-season competitiveness may possibly suggest a fitness cost endowed by glyphosate resistance alleles in this weed species^23–25^. This fitness study was conducted in the absence of crop (fallow); however, results might not be very different in the presence of sugar beet which is among the least competitive crops against *B. scoparia*^9–11^. It is also acknowledged that fitness outcomes may vary depending on the presence and extent of crop competition (e.g. *B. scoparia* would be less competitive in the presence of cereals or corn compared to fallow or sugar beet)^25,27^.

Results from this research may aid in predicting the evolutionary trajectory of GR *B. scoparia* under field conditions with the continued use of glyphosate. This is more important to understand when there is a lack of other effective sites-of-action herbicides to control this weed in sugar beet. This is the first study to report the fitness traits of GR *B. scoparia* with low to high levels of resistance (2 to ≥ 8 *EPSPS* gene copies) in the presence of varying glyphosate rates in the field. Irrespective of the total glyphosate rate, the variation in *EPSPS* gene copy number had no effect on the fitness traits except seed production in GR *B. scoparia*. Sequential applications of glyphosate with a total rate of 2214 g ae ha^−1^ or higher prevented seed production of GR *B. scoparia* with low and moderate glyphosate resistance (2–4 and 5–6 *EPSPS* gene copies, respectively), implying the importance of timely, sequential applications of glyphosate at full recommended rates in GR sugar beet. However, the highly resistant *B. scoparia* with ≥ 8 *EPSPS* gene copies surviving four sequential applications of glyphosate (total rate of 3,954 g ae ha^−1^) should be manually removed before they flower (to prevent gene flow through pollen or seeds) in GR sugar beet fields. More importantly, the results indicate that offspring from glyphosate survivors may increase the probability of genotypes with increased *EPSPS* gene copy numbers in a field population of *B. scoparia*.

A zero-tolerance approach against seed production from GR *B. scoparia* plants should be adopted in GR sugar beet fields. Sugar beet growers should adopt an integrated weed management (IWM) program to manage GR *B. scoparia*. This includes the use of mechanical and ecological approaches such as tillage, cover crops, and diverse crop rotations that employ different cultural management practices (planting dates, harvest dates, and crop canopy) and effective alternative herbicides other than glyphosate (in rotational crops) to reduce the frequency of GR *B. scoparia* on their sugar beet fields^27^. Selecting the most competitive crops such as cereals or corn in rotation with sugar beet will be important in reducing seed inputs of *B. scoparia*^27,28^. This will also provide an opportunity to reduce kochia seed banks being exposed to glyphosate, thereby slowing down the evolution and spread of glyphosate resistance. Selection of a late-planted crop grown in the U.S. Great Plains such as dry beans, in a diverse crop rotation plan could also reduce herbicide exposures through non-chemical control tactics such as stale seedbed^27^. These IWM tactics would be more important for a weed like *B. scoparia* with a short-lived seed^2,3^.

Future studies on understanding the fitness attributes of glyphosate resistance alleles in *B. scoparia* under varied biotic and abiotic stresses (herbivory, disease, and crop competition) will further help to validate these results and aid in developing long-term IWM strategies in sugar beet-based crop rotations of the U.S. Great Plains. In addition, future research should assess the frequency of GR resistance alleles of *B. scoparia* over multiple years under diverse management practices in crop production fields.

## Methods

### Seed source

Seeds of a segregating GR *B. scoparia* population identified from a wheat field (45°54′54.76″N; 108°14′44.15″W) in 2013 in Hill County, Montana, USA (designated as MT009) were used. The field was under a continuous no-till wheat-fallow rotation for > 8 years and had a history of repeated glyphosate use (at least 3 applications per year) for weed control during the summer fallow phase prior to winter wheat planting. The permission of land owner was obtained prior to *B. scoparia* seed collection. All experimental research and field studies on plants, including the collection of plant material complied with the Montana State University guidelines and state/US legislation. Seeds of the field-collected population were used to generate GS and GR *B. scoparia* subpopulations through recurrent group selection procedure as described below.

### Development of GS and GR subpopulations

Field collected seeds of MT009 population were sown on the surface of plastic trays (53 by 35 by 10 cm) filled with commercial potting soil (VERMISOIL, Vermicrop Organics, 4265 Duluth Avenue, Rocklin, CA, USA) in a greenhouse in the fall of 2013 at the Montana State University Southern Agricultural Research Center (MSU-SARC) near Huntley, MT, USA. Growth conditions in greenhouse were maintained at 25/22 ± 2 °C day/night temperatures and 16/8 h day/night photoperiods supplemented with metal halide lamps (450 μmol m^-2^ s^-1^). After emergence, approximately 200 uniform seedlings were individually transplanted in plastic pots (10-cm diam) containing the same potting mixture and grown for 6 weeks. A set of three clones (3 shoot cuttings) from each plant were then prepared and transplanted in plastic pots (10-cm diam) as described by Kumar and Jha^22^. At the 8- to 10-cm height, all cloned seedlings were separately treated with 435 (0.5×), 870 (1×), and 1740 (2×) g ae ha^−1^ of glyphosate (Roundup Powermax, Bayer Crop Science, Saint Louis, MO, USA) where 1× = field-use rate of glyphosate. All three glyphosate treatments included ammonium sulfate (2% w/v). Glyphosate applications were made using a cabinet spray chamber (Research Track Sprayer, De Vries Manufacturing, RR 1 Box 184, Hollandale, MN, USA) equipped with an even flat-fan nozzle tip (Teejet 8001EXR, Spraying System Co., Wheaton, IL, USA), calibrated to deliver 140 L ha^−1^ of spray solution at 276 kPa. Treated seedlings were returned to the greenhouse, watered as needed, and fertilized [Miracle-Gro water soluble fertilizer (24-8-16), Scotts Miracle-Gro Products Inc., 14111 Scottslawn Road, Marysville, OH, USA] bi-weekly to maintain good plant growth. At 21 days after treatment, clones surviving the 2× rate of glyphosate were considered as ‘glyphosate-resistant (GR)’ and the clones that did not survive 1× rate of glyphosate were considered as ‘glyphosate-susceptible (GS)’. The parent *B. scoparia* plants corresponding to survived (resistant) or not-survived (susceptible) clones were transplanted separately in 20-L plastic pots (group of 3 to 4 plants pot^−1^) containing same potting soil for seed production. All 3- to 4 plants in each pot were collectively covered with a single pollination bag (DelStar Technologies, Inc., 601 Industrial drive, Middletown, DE, USA) prior to flower initiation to restrict cross-pollination between GR and GS plants. At maturity, seeds from the respective GR and GS parent plants were collected and cleaned separately using an air column blower. The collected seeds from GR plants were subjected to three generations of recurrent group selection with the 2× rate of glyphosate in each generation. Seeds of GS plants were also subjected to recurrent group selection for three generations without glyphosate. Progenies of the GS plants were grown and sprayed with 1× rate of glyphosate to confirm the susceptibility to glyphosate in each generation^23^. This procedure allowed the development of relatively genetically homogenous GR and GS subpopulations from within a single *B. scoparia* population.

### Determination of *EPSPS* gene copy number

Previously established protocols were adopted to estimate the relative *EPSPS* gene copy number in seedlings of GR and GS subpopulations through quantitative real-time polymerase chain reaction (qPCR)^16–18^. The *ALS* gene was used as reference since the relative *ALS* gene copy number and transcript abundance did not vary across *B. scoparia* samples^17,18,29^. Relative *EPSPS:ALS* gene copy number is a ratio of *EPSPS* to *ALS* PCR product fluorescence. Due to small differences in amplicon size, qPCR run conditions, and fluorescence detection, the values presented were estimates of relative gene copy number^29^.

A total of 600 seedlings from the GR (450 seedlings) and GS (150 seedlings) *B. scoparia* subpopulations (developed by recurrent group selection) were grown in a greenhouse at MSU-SARC near Huntley, MT, USA in 2015 and 2016 to select enough plants for the field study each year. At 4-to 6-cm height, young leaf tissues (100 mg) from each seedling were sampled, frozen with liquid nitrogen and ground into powder using mortar and pestle. Genomic DNA were extracted from the tissue samples using the protocol from Qiagen Dneasy plant mini kit (Qiagen Inc., Valencia, CA, USA). Genomic DNA quantity and quality were determined using a Smartspec Plus spectrophotometer (Bio-Rad Company, CA, USA) and gel electrophoresis with 1% agarose, respectively. High quality genomic DNA (260/280 ratio of ≥ 1.8) were used to determine the relative *EPSPS* gene copy number. Two sets of primers to amplify the *EPSPS* and *ALS* genes, the final reaction volume and reagents used for each qPCR reaction, and the qPCR conditions used in this study were the same as previously described by Kumar and Jha^22^. Each qPCR reaction was performed on a Bio-Rad 96-well PCR plate in triplicates and fluorescence was detected using CFX Connect Real-Time PCR detection system. A negative control consisting of 250 nM of each forward and reverse primer, 1× Perfecta SYBR Green supermix, and deionized water with no DNA template was included. The *EPSPS* genomic copy number relative to *ALS* gene was estimated by ΔC_T_ method (ΔC_T_ = C_T_, *ALS*-C_T_, *EPSPS*)^18,29^. The relative increase in the *EPSPS* gene copy number was calculated as 2^ΔCT^.

### Survival and fecundity traits of GR and GS *B. scoparia* subpopulations

Seedlings (4- to 6-cm tall) of GR and GS *B. scoparia* subpopulations with known *EPSPS* gene copy numbers were transplanted into a fallow field in the summer of 2015 and 2016 at the MSU-SARC near Huntley, MT, USA. All transplanted *B. scoparia* seedlings were equally spaced at 1.5 m apart from each other and all plants were fertilized biweekly [2 to 3 g of MIRACLE-GRO water soluble fertilizer (24-8-16)] and irrigated as and when needed to avoid moisture stress. Experiments were conducted with a factorial arrangement of treatments (Factor A and Factor B) in a randomized complete block design, with 6 replications. Each transplanted *B. scoparia* seedling was an experimental unit. The factor A (4 levels) was comprised of *B. scoparia* plants with 1, 2–4, 5–6, and ≥ 8 *EPSPS* gene copy numbers, which were categorized as susceptible, low, moderate, and highly resistant plants, respectively based on their percent visible injury response to glyphosate. The factor B (ten levels) was comprised of increasing rates of glyphosate applied as single or sequential applications. Current labels of glyphosate allow a total of 3954 g ae ha^−1^ in split POST applications in GR sugar beet. As per the label, the maximum glyphosate rate of 2214 g ae ha^−1^ is allowed from crop emergence to 8-leaf stage of sugar beet and 1740 g ae ha^−1^ of glyphosate from 8-leaf stage to canopy closure or 30 days prior to sugar beet harvest. Hence, the tested total glyphosate rates were 0, 108, 217, 435, 870, 1265, 1740 [870 followed by ( +) 870], 2214 [1265 + 949], 3084 [1265 + 949 + 870], and 3954 [1265 + 949 + 870 + 870] g ae ha^−1^ along with ammonium sulfate (2% w/v). Sequential applications were made at 7- to 14-day intervals, with first application at 8- to 10-cm tall *B. scoparia* seedlings using a CO_2_-operated backpack sprayer fitted with a single AIXR 8001 flat-fan nozzle calibrated to deliver 94 L ha^−1^. Glyphosate rates and applications timings were selected to simulate the 2-leaf, 6-leaf, 8–10 leaf, and the canopy closure stage of GR sugar beet.

### Data collection

Percent visible control (relative to the non-treated) on a scale of 0 to 100 (0 means no control and 100 means complete plant death^30^) for each individual plant (240 plants total each year) were assessed at 7, 14, and 21 days after glyphosate treatment. Data on number of days from transplanting to 50% flowering (half of the inflorescences from each plant were covered with visible flowers) and seed set (seeds on half of the inflorescences from each plant were turned brown) were recorded for an individual plant. Each plant was covered with a pollination bag (DelStar Technologies, Inc., 601 Industrial drive, Middletown, DE, USA) prior to flowering to prevent any cross-pollination. At the time of flowering, pollens from each survived plant were collected in early morning hours (between 8 to 10 am). At maturity, each individual plant was harvested and threshed to determine 1000-seed weight and seeds plant^−1^.

### Pollen and progeny seed viability

Pollens and seeds collected from individual *B. scoparia* plants (240 plants total each year) were tested for viability using a tetrazolium test. Pollens were collected in petri dishes by shaking the whole plant at the time of flowering. Four sub-samples of pollens from each petri dish were transferred into glass slides. The pollens in the glass slides were soaked with a tetrazolium chloride solution (10 g L^−1^), sealed with a cover slip using a nail polish and were incubated at room temperature for an hour. Viable (red) and non-viable pollens (yellow/white) were counted using a simple microscope. The physical structure of viable and non-viable pollens was also checked for any deformity using a compound microscope. Pollen viability for individual plants (240 plants total each year) was calculated as percent viable pollens of the total number of pollens counted.

For seed viability test, twenty-five intact seeds collected from each individual plant (240 plants total each year) from the field were evenly placed in between two layers of filter papers (WHATMAN Grade 2, SigmaAldrich, St Louis, MO, USA) inside a 10-cm-diameter petri dish. Seeds were soaked with a 5-ml of distilled water and the filter papers were kept moist for the entire duration of the germination test. Light is not required for *B. scoparia* seed germination^31^, so the petri dishes were wrapped with a thin aluminum foil and placed inside an incubator (VMR International, Sheldon Manufacturing, Cornelius, OR, USA) with alternating day/night temperatures set to 20/25 °C^23^. Seeds with a visible uncoiled radicle tip longer than the seed diameter was considered germinated^32,33^. Radicle length was measured from three randomly selected germinated seeds 24 h after incubation to test the seedling vigor. The number of germinated seeds in each petri dish were counted daily until no further germination was observed for 10 consecutive days. Non-germinated seeds were tested for viability by soaking the seeds with tetrazolium chloride solution (10 g L^−1^) for 24 h^23,34^. Seeds with a red-stained embryo examined under a dissecting microscope (tenfold magnification) were considered viable^35^. Seed viability was expressed as the percentage of total viable seeds.

### Relative fitness (*w*)

Fitness is the evolutionary potential for success of a genotype based on survival, competitive ability, and reproduction. Individuals with the greatest number of offspring and with the most genes contributing to the gene pool of a population are considered most fit genotypes^36^. Fitness of a genotype is determined by comparison of its vigor, productivity or competitiveness relative to the other genotype by quantifying specific traits such as seed dormancy, flowering date, seedling vigor, seed production, and other factors that can possibly influence the survival and reproductive success of a genotype^36,37^. In this study, relative fitness (*w*) of GR *B. scoparia* was calculated as the reproductive rate (seed production plant^−1^) of a resistant genotype (*B. scoparia* plants with 2–4, 5–6, and ≥ 8 *EPSPS* gene copies) relative to the maximum reproductive rate of the susceptible genotype (*B. scoparia* plants with 1 *EPSPS* gene copy) in the population. The relative fitness (*w*) of susceptible plants was assumed to be one.

### Statistical analyses

A natural logarithm transformation was performed on data for time to 50% flowering, time to seed set, seeds plant^−1^. An arcsine square root transformation was performed on data for pollen viability, visible control, seed viability, and relative fitness (*w*) before subjecting to analysis of variance, however all data were presented in their back-transformed values. No transformation was needed for 1000-seed weight and radicle length data. Experimental year, *B. scoparia* plants with different *EPSPS* copy number groups, glyphosate rate, and their interactions were considered fixed effects and replication nested within a year was considered as a random effect in the model. Data on percent visible control, time to 50% flowering, pollen viability, time to seed set, 1000-seed weight, and seeds plant^−1^, seed viability and radicle length were subjected to ANOVA using Proc Mixed in SAS (SAS version 9.4, SAS Institute, Cary, NC, USA) to test the significance of experimental run, treatment factors, and interactions. The ANOVA assumptions for normality of residuals and homogeneity of variance were tested using Proc Univariate and PROC GLM in SAS. Means were separated using Tukey–Kramer’s HSD with α = 0.05. Furthermore, data on percent visible control and seeds plant^-1^ for each group of *B. scoparia* plants with different *EPSPS* gene copy number were regressed against total glyphosate rates using a four-parameter log-logistic model Eq. (1)^38,39^:

1 $$Y=c+\{d-c/\{1+\mathrm{exp}[b\left(\mathrm{log}\left(x\right)-\mathrm{log}\left(ED50\right)\right)]\}$$

where *Y* is the percent visible control or seed production plant^−1^ (% of nontreated); *d* is the upper asymptote (the highest estimated % control or % seed reduction); *c* is the lower asymptote (the lowest estimated % control or % seed reduction); *ED*_50_ is the effective rate of glyphosate needed to achieve 50% control or 50% reduction in seed production; and *b* denotes the slope around the inflection point “*ED*_50_.” Slope parameter (*b*) indicates the response rate of each group of *B. scoparia* plants with different *EPSPS* gene copy number to glyphosate rates (i.e., a slope with a large negative value suggests a rapid response of selected *B. scoparia* group). The Akaike Information Criterion (AIC) was used to select the nonlinear four-parameter model. A lack-of-fit test (*P* > 0.10) was used to confirm that the nonlinear regression model Eq. (1) described the response data for each *B. scoparia* group^38^. Parameter estimates, *ED*_90_, and *SR*_99_ values (i.e. effective rate required for 90% control or effective rate required for 99% reduction in seed production) for each group of *B. scoparia* plants with different *EPSPS* gene copy number were determined using the ‘*drc*’ package in *R* software^37,39^. Parameter estimates of *B. scoparia* groups were compared using the approximate *t*-test with the ‘*compParm*’ and ‘*EDcomp*’ functions in the ‘*drc*’ package of the *R* software^39,40^.

## Data Availability

The datasets generated and/or analyzed during the current study are available from the corresponding author on reasonable request.
